# Theory of Mind, Self-Perceptions, and Peer Popularity in Middle Childhood and Early Adolescence

**DOI:** 10.3390/children12030281

**Published:** 2025-02-25

**Authors:** Christopher Osterhaus, Giulio D’Urso, Susanne Koerber, Sandra L. Bosacki

**Affiliations:** 1 Developmental Psychology in Education, University of Vechta, 49377 Vechta, Germany; 2 Department of Law, Economics, and Human Sciences, “Mediterranea” University of Reggio Calabria, 89124 Reggio Calabria, Italy; giulio.durso@unirc.it; 3 Department of Early Childhood Education, Freiburg University of Education, 79117 Freiburg, Germany; susanne.koerber@ph-freiburg.de; 4 Department of Educational Studies, Brock University, St. Catharines, ON L2S 3A1, Canada; sandra.bosacki@brocku.ca

**Keywords:** theory of mind, peer popularity, self-perceptions, middle childhood, adolescence

## Abstract

**Background/Objectives:** Peer popularity is often associated with children’s and adolescents’ Theory of Mind (ToM), as well as with self-perceptions. This paper describes two cross-sectional studies that investigate the individual differences and associations among peer popularity, ToM, and self-perceptions (i.e., several dimensions of self-esteem, including global, academic, or physical self-esteem). **Methods:** Study 1 involved 229 German children aged 5 to 8 years. Study 2 involved 127 Canadian adolescents aged 10 to 12 years. Participants in both studies completed measures of ToM, self-perceptions, and peer popularity. **Results:** Study 1 found significant associations among children’s ToM and self-perceptions (their global self-esteem) but found no associations with children’s peer popularity. Study 2 revealed significant positive associations between adolescents’ ToM and their peer popularity, as well as between ToM and self-perceptions (in particular, adolescents’ academic self-esteem). **Conclusions:** Our findings support the view that ToM matters for children’s and young adolescents’ self-perceptions and plays an increasingly important role in their everyday social life. Curricular implications for ToM, identity formation (self-perceptions), and peer relations are discussed.

## 1. Introduction

Understanding what other people want, feel, or believe is an essential mentalizing skill that helps children and adults navigate their social lives [1,2]. Theory of Mind (ToM) is defined as the ability to see oneself and others in terms of mental states, and as such, it includes the ability to recognize one’s own and others’ desires, emotions, beliefs, and intentions [3]. Decades of research show that during the preschool years, children acquire the understanding that people’s actions are guided by mental states, which cannot be directly observed [4].

Despite this history of research, gaps remain in the development of ToM within a social realm, especially during middle childhood and early adolescence [5]. Building on the foundational ToM skills that are acquired in preschool years, mentalizing skills develop in complexity as advanced ToM becomes increasingly multidimensional and specified [6]. That is, with maturity, ToM continues to develop, and children begin to acquire more-advanced theories of mind, allowing them to detect more subtle cues about the contents of others’ minds during more complex and ambiguous social settings [5,7,8]. For instance, Osterhaus and Koerber [9] showed that 6- to 10-year-old children engage in more-complex social reasoning and understand why others sometimes do not follow social convention or unintentionally say or do things that may be perceived as socially inappropriate or awkward (i.e., commit acts of faux pas). Older children also further develop the ability to reason about social and moral dilemmas [10]. Put differently, during middle childhood and adolescence, children acquire a clearer understanding of what they themselves and others desire, feel, and believe, and they better understand their own minds.

### 1.1. ToM and Self-Perceptions

Research on self-concept, and in general self-perceptions, remains a key area of interest within psychology and education, particularly during times when the self is in major transformation as it is in middle childhood and early adolescence. Self-perceptions refer to the (conscious) beliefs that people have about themselves, which are mostly self-representations of the attributes and characteristics of one’s self [11].

A central aspect of people’s self-perceptions is their self-concept. The “theory” view of self-concept [12] assumes that the conceptual self is a multidimensional cognitive representation or theoretical framework that is based on how individuals understand their own beliefs, desires, and intentions [13]. Following Higgins [14], we consider self-esteem to be an evaluation, or judgment of the self-concept, as well as an association between the real self and the ideal self. In this sense, self-esteem is evaluative, self-reflective, and self-judgmental [11].

Self-esteem and a strong sense of identity within social interactions [1] are—due to their self-reflective character—products of evaluative processes that we assume to be linked to ToM skills such as emotion recognition and perspective-taking. In particular, ToM skills could serve as useful tools to help a young person develop a strong sense of identity and self-esteem by enabling them to more clearly perceive and distinguish perceptions of the real self and the ideal self. That is, such mental tools could help children and young adolescents to balance the characteristics and qualities that they ascribe to themselves (i.e., the real self), along with the internal representation of the moral standards and norms imposed by society that children believe they ought to possess (i.e., the ideal self).

Few studies explicitly explore how ToM relates to self-perceptions in children and adolescents [15]. The few studies that exist reveal mixed findings, and only some underline the link between ToM and self-perceptions [16,17,18], showing that more-advanced ToM skills are indeed associated with higher self-esteem. Evidence from neuroscience studies further lends support to the claim that ToM and self-esteem are associated as they show that the same region, the medial prefrontal cortex (mPFC), guides the self-referential processes that compare oneself to others and help one to understand one’s own and others’ perspectives [19].

### 1.2. ToM and Peer Popularity

Peer popularity refers to a child’s social status within their peer group and is often distinguished from peer acceptance. While peer acceptance reflects how well-liked a child is, popularity specifically refers to a child’s visibility, social dominance, and influence within the peer network [20], and it is a construct that has far-reaching consequences, including effects on children’s academic performance and their academic achievement goals [21]. Middle childhood and early adolescence are times when peer relations increase in importance and complexity and when children learn more and more to think about their interactions and status with their peers [22,23]. Especially during elementary school, children begin to think differently and to care more about how they are perceived by their peers in terms of popularity and how much they are liked by others [24].

How much children are liked by others and how popular they are is often measured with a peer-rated sociogram. This prominent, widely accepted measure asks either children within a classroom to define those (three) children with whom they like to play the most and/or vice versa the least [25], or to evaluate each classmate on a Likert scale with respect to how much they would enjoy spending time with them [26,27,28].

Studies show that children who are perceived by their peers to be popular generally possess a positive attitude, are often in demand by their peers to play with, and are characterized as strong leaders and generous or likely to share [29]. Furthermore, there is evidence that those youth rated as popular by their peers generally show a higher level of socialization and greater cognitive abilities than peers not belonging to this social status, as well as a minimal level of aggression and social isolation [30]. Therefore, it is assumed that peer popularity is associated with ToM, because having a good understanding of ourselves and others’ mental states helps us to build better relationships and to be well liked.

In recent years, various studies investigated the relation between ToM and peer popularity, revealing mixed results, e.g., [31]. A meta-analysis of 20 studies (including over 2000 children), however, resolved these seemingly contradictory findings: Slaughter and colleagues [20] showed that high levels of ToM are indeed related to high levels of peer popularity, and they found that the seemingly contradictory results stem from an overall small effect for the association between ToM and peer popularity (children’s ToM accounted for only 3.6% of the variance in peer popularity).

Despite these research findings with, on the one hand, associations between ToM and peer popularity [20] and ToM and self-perceptions on the other hand, few studies exist that assess the links among ToM, self-perceptions, and peer popularity in older childhood and early adolescence within a single study.

### 1.3. The Present Study

The school classroom provides a potentially rich context within which to investigate ToM, self-perceptions, and peer popularity, and this study aims to explore the connections among school-aged children’s and young adolescents’ mindreading skills, self-perceptions, and peer popularity. Specifically, although past work has shown that the ability to take the perspectives of others and self-perceptions are linked to peer ratings of popularity [32], fewer studies explore if ToM skills influence one’s self-view and popularity within the peer group. To the best of our knowledge, to date, researchers have yet to explore these constructs in a single study and control for the mutual associations between the variables. To address this gap in the literature, the two present studies explore (1) if links exist between ToM, self-perceptions, and peer popularity, and (2) if these are uniform across development (i.e., in middle childhood and early adolescence). We hypothesize that ToM, or the ability to make sense of people’s behaviors in terms of their mental states, helps to shape how a person makes sense of the minds and emotions of both self and within a social context [33]. Study 1 addresses this issue in children aged 5 to 8 years; Study 2 investigates the potential associations between ToM, self-esteem, and peer popularity in young adolescents aged 10 to 12 years. Both studies expand the existing literature by examining two target groups and focusing on the developmental perspectives of key age groups (middle childhood and early adolescents) regarding the role of ToM in the participants’ social experiences.

## 2. Study 1

### 2.1. Materials and Methods

#### 2.1.1. Participants

The participants were 229 children aged 5 to 8 years. Specifically, there were 67 five-year-olds (*M* = 5.17 years; *SD* = 3 months; 38 girls, 29 boys), 97 seven-year-olds (*M* = 6.92, *SD* = 5 months; 48 girls, 49 boys), and 65 eight-year-olds (*M* = 7.92; *SD* = 5 months; 37 girls, 28 boys). The children were recruited from nine kindergartens and six elementary schools in or near a midsized city in southern Germany. Written consent was obtained from the parents for all children. Data from this study have been published in [18,34]. Ethics and legal approval were provided by the District Government. Recommendations by professional organizations, such as the American Psychological Association (APA) and the German Association for Psychology (DGPs), were followed to implement appropriate safeguards. These included obtaining informed consent from all participants (or their guardians), ensuring anonymity and confidentiality in data collection and reporting, and minimizing any potential risks to participants’ well-being. Additionally, participation was entirely voluntary, with the option to withdraw at any time without consequence.

#### 2.1.2. Measures

First-order ToM. We used a German translation [35] of the Wellman scale [36] to assess children’s first-order ToM. This battery comprises 6 tasks, which assess whether children understand that (1) people can have diverse desires, (2) that they can hold diverse beliefs, (3) that they can have different access to knowledge, (4–5) that they may believe something that is not true, and (6) that they can hide their true emotions.

Advanced ToM (see Table 1). We used an advanced ToM battery [34,37] to assess children’s complex mindreading skills across three factors: (1) social reasoning; (2) reasoning about ambiguity; and (3) recognizing transgressions of social norms (see Table 1). The battery comprises 18 widely used measures, including measures of higher-order false belief understanding [3,38,39], understanding of nonliteral speech [40], and emotion and mental state recognition from the eyes [41] (all social reasoning), as well as measures of understanding multiple perspectives [42] (reasoning about ambiguity), and the recognition of faux pas [43] (recognizing transgressions of social norms). Ten tasks were administered in individual interviews; eight were presented in a paper-and-pencil format in either small-group tests (in elementary schools) or individual interviews (in kindergarten).

To make the Eyes Test [41] easier for our young sample, we presented the children with short statements in the answer options that explained the mental state term (e.g., ‘he is being nice’) that was presented in the photograph (e.g., friendly).

Self-perceptions. We used a 7-item scale [44] to measure children’s self-esteem. The 7 items assessed children’s feelings about themselves regarding their physical appearance, as well as cognitive, social, or scholastic competencies. Children rated these statements (e.g., ‘When I see a picture of myself, I feel…’) on a 5-point Likert scale [from 0 (bad) to 4 (good)]. The reliability of the scale was acceptable, with Cronbach’s α = 0.63.

Peer popularity. An estimate of children’s peer popularity was obtained by asking all children to nominate the three children with whom they liked to play the most, and the three children with whom they liked to play the least [25]. Based on their peer nominations, we calculated, for each child, a popularity score (i.e., the times a child was among the top three favorite children that others play with) and unpopularity score (i.e., the times a child was among the top three least favorite children). Based on these scores, as well as per classroom, we created z scores that considered the different sizes of the individual classrooms. We used the popularity score in our analysis.

### 2.2. Results

The descriptive results are given in Table 2. There were significant differences for all three advanced ToM factors between kindergarten and Grade 2. Children’s average reported self-esteem was relatively high (*M* = 3.14 across all grades); nonetheless, there was a significant developmental trend, with scores being higher in older children. Children’s popularity and unpopularity scores did not differ between the grades. Also, there were no significant gender differences with respect to any of the variables assessed: first-order ToM [*F*(1, 227) = 0.306, *p* = 0.581], social reasoning [*F*(1, 227) = 0.000, *p* = 0.992], reasoning about ambiguity [*F*(1, 227) = 0.116, *p* = 0.734], recognizing transgressions of social norms [*F*(1, 227) = 1.826, *p* = 0.178], self-esteem [*F*(1, 227) = 1.252, *p* = 0.264], and popularity [*F*(1, 227) = 0.252, *p* = 0.616].

The correlations between all measures are given in Table 3. There were significant, but weak correlations between all three advanced ToM factors. Children’s social reasoning was associated with their self-esteem (*r* = 0.170, *p* = 0.011). There were no significant correlations between children’s popularity and any of (advanced) ToM, including the first-order scale.

## 3. Study 2

### 3.1. Methods

#### 3.1.1. Participants

The participants were 127 elementary grade school children (70 girls; 57 boys) for whom data on peer popularity was obtained within a larger study on adolescents’ social and self-cognitions and peer relations. There were 48 students in Grade 4 (25 girls, 23 boys; *M* = 9;7), 34 students in Grade 5 (20 girls, 14 boys; *M* = 10;6), and 45 students in Grade 6 (25 boys, 20 girls; *M* = 11;6). The students came from 3 schools within a school board situated in a mainly Euro-Canadian, middle SES, mid-sized Southern Ontario city. All students reported English as their first language (100%). Parents’ informed consent and child assent were obtained for all participants. In particular, consent forms for participation were distributed to all parents and/or legal guardians and signed. Ethical approval was obtained from the Institutional Review Board at Brock University.

#### 3.1.2. Measures

Advanced ToM (see Table 1). To assess children’s ToM, we used two brief vignettes from the socially ambiguous stories (SAS) [19,45]: one involving three girls and the other story involving three boys. The SAS assess students’ mentalizing skill involved in the interpretation of social meaning from ambiguous stories within a social context. For instance, in one story (Nany/Margie), children are told about two girl friends who walk towards a new girl in the playground. As the two friends approach the new girl, the new girl wonders what the two girls (who are unfamiliar to her as she is new to school) want. Each story was followed by four sets of questions that represent the constructs of advanced ToM including: (1) conceptual perspective-taking or the understanding of higher-order mental states (e.g., ‘Why did Nancy smile at Margie?’), (2) empathetic sensitivity (e.g., ‘How do you think the new girl feels and why?’), (3) person perception (‘Choose a character in the story and describe her. What kind of person do you think she is?’), and (4) alternative thinking (‘Is there another way to think about this story [yes/no], If yes, how?’) (see [19] for further details). To control for memory and comprehension effects, two comprehension questions preceded the story questions. If the comprehension questions were answered incorrectly, the researcher re-read the story. Finally, to control for order effects, the two stories were read to participants in counterbalanced order.

Zero points were given for “I don’t know”/no answer or tangential responses, 1 point for responses that included behavioral or situational descriptions, 2 points for responses that included mental states, or acts of communication or perception, and 3 points for responses that included an integration of two or more mental states and related them to each other in a coherent manner such as moral judgements or recursive psychological statements. Each response was given one score only, so that participants were given credit for their “best” answer. Thus, the total score represented a best estimate of the participants’ ability to understand social situations. The items of each story were summed resulting in the maximum total score of 21 for each story (Conceptual Role-Taking—range of scores 0–9, Empathic Sensitivity—range 0–6, Person Perception—range 0–3, Alternative Ending—range 0–3, Total for each story = 21), with a high score representing a more sophisticated understanding of mental states and feelings (i.e., a more complex ToM). Interrater reliability (calculated with 38% of the transcripts) was high, with Cohen’s kappa for the girl story at 0.98 (range from 0.90–1.00) and for the boy story at 0.99 (range 0.95–1.00). Internal consistency was lower, with Cronbach’s α for the Nancy/Margie story and Kenny/Mark story at 0.67 and 0.69, respectively. A total ToM score was calculated from the sum of the two story-totals (maximum total score of 42 [21] points per story).

Peer popularity. Peer popularity was measured with a peer-rated sociogram, which is considered the least problematic and most appropriate method of assessing peer status [26,27]. Each participant was provided a class list and then rated each of their classmates on a 5-point scale as to how much she or he would enjoy spending time with that person. In the analysis, z-scores were used.

Self-Perceptions. To assess students’ self-esteem, we used Harter’s Self-Perception Profile for Children (SPPC) [46]. This 36-item, self-report questionnaire was administered in class according to standardized procedures, and it assessed self-perceptions on a 4-point Likert scale in 6 self-dimensions including (1) social, (2) academic, (3) physical appearance, (4) behavioral conduct, (5) athletic competence, and (6) global self-worth. The SPPC is frequently used in research with children and young adolescents, e.g., [14], with internal consistency reliabilities for all sub-scales ranging between 0.71 and 0.84 (Cronbach’s α) [46] and test–retest reliability (0.69–0.87, varying with subscale). The reliability for the entire scale was excellent, with Cronbach’s α = 0.96. Subscale reliabilities were good, ranging between Cronbach’s α = 0.76 (social acceptance) and 0.84 (academic competence).

### 3.2. Results

Table 4 shows the means and standard deviations of the scores. There were no significant differences between the grades for any of the variables, including ToM, popularity and the distinct dimensions of children’s self-esteem. With the exception of the subaspect “behavioral conduct” of the SPPC, for which girls reported a higher score (*M* = 19.46, *SD* = 3.79) than boys (*M* = 17.33, *SD* = 4.45) [*F*(1, 122) = 7.397, *p* = 0.007], there were no gender differences: ToM [*F*(1, 126) = 0.001, *p* = 0.977], social [*F*(1, 122) = 0.361, *p* = 0.549], academic [*F*(1, 122) = 0.027, *p* = 0.870], physical [*F*(1, 122) = 3.269, *p* = 0.073], athletic [*F*(1, 122) = 0.717, *p* = 0.399], and global self-worth [*F*(1, 121) = 0.001, *p* = 0.974], as well as peer popularity [*F*(1, 125) = 1.104, *p* = 0.295].

The correlations between all measures are given in Table 5. There were significant associations between all subaspects of the SPPC (self-esteem), ranging between 0.15 and 0.71. ToM was related to only self-ratings of academic self-esteem (*r* = 0.182, *p* = 0.043) and other ratings of popularity (*r* = 0.22, *p* = 0.011). Peer popularity did not correlate with any aspect of the SPPC.

We tested two saturated models in Mplus version 8. In the first model, we tested if ToM and students’ academic self-worth (which was the only aspect of the SPPC correlated with ToM) were related to popularity, considering gender and grade as control variables. In the second model, we tested the interaction terms between ToM and academic self-worth in predicting popularity, controlling again for gender and grade. An overview of the models is given in Table 6. In line with the correlational analysis, our findings show that only ToM is connected to peer popularity (Model 1: b = 0.24, *p* < 0.01). There was no interaction between ToM and academic self-esteem in the prediction of peer popularity (b = −0.12; n.s.).

## 4. Discussion

What are the associations between ToM, self-perceptions, and peer popularity across middle childhood and early adolescence? This was the main question addressed in the present study, which showed a significant link between ToM and self-perceptions across two independent samples from two different countries of children in middle childhood (aged 5 to 8 years) and early adolescence (aged 10 to 12 years). Peer popularity was related to ToM only in the older but not younger sample, and no significant associations emerged between peer popularity and children’s self-perceptions. This is in line with several studies conducted in Germany (the setting of Study 1) and Canada (the setting of Study 2) which show that children’s and adolescents’ self-perceptions relate to ToM [19,20,21] based on the hypothesis that ToM enables one to recognize mental states in others and in oneself.

Consistent with this hypothesis, our findings show a significant association between ToM and global self-esteem in middle childhood (Study 1), as well as between ToM and academic self-esteem in early adolescence (Study 2). It is worth noting that academic self-esteem was the only aspect of young adolescents’ self-perceptions that was correlated with ToM. All other dimensions of the self, including social, athletic, physical, behavioral-conduct, and global self-esteem, were not associated with ToM. Such findings could suggest that aspects of the self, such as athletic or physical self-esteem, may not strongly rely on the mentalizing skills because they can be assessed rather objectively by comparing oneself and one’s physical attributes to others [10].

Thus, it is not surprising that these dimensions of the adolescents’ self-perceptions did not correlate with ToM. What is however surprising is that we found no connections between young adolescents’ social self-esteem, or how they perceived themselves to be socially accepted, and their ToM. However, our results showed that higher levels of ToM related to higher perceptions of one’s scholastic or academic self, or how adolescents viewed themselves as strong scholars. Such findings contradict past studies [1] and suggest that academic self-esteem may require children and young adolescents to carefully read the mental states of their teachers to receive feedback about their performance and self-esteem as students. In support of Higgins’ self-concept theory [14], ToM could therefore contribute to creating a real self, congruous to adolescents’ role as students, because it modulates their internal world in relation to their own self with the evolved needs that are typical in the developmental period of early adolescence. Research shows that children with strong ToM skills are also more sensitive to teacher criticism [47], which mitigates the positive association between ToM and academic performance, which in turn may give children and young adolescents more informative feedback about their academic performance.

Peer popularity has been linked to ToM across several studies, including a meta-analysis [20], which reported a small effect for the association between ToM and peer popularity in middle childhood and early elementary school. In the present study, we found no significant association between ToM and peer popularity in our middle-childhood sample, but only a significant effect in the sample of young adolescents. In early adolescence, ToM could contribute to creating a wealth of socio-emotional skills useful for negotiating and building friendships with peers and, consequently, for being more popular. In addition, given the possible reciprocal relations between peer popularity and ToM in that social interactions may promote mindreading skills [26], those youth who are perceived as popular by their friends may have increased interaction with their friends, which in turn may provide more opportunities to engage in social communication and engage in perspective-taking and emotion recognition. Such complex relations suggest the need for further study on the links between dimensions of self-concept and perceptions of popularity and social competence among adolescents.

The former finding in younger children suggests also that ToM skills such as emotion recognition and perspective-taking may be a less relevant predictor of peer popularity in younger children. Younger children typically hold more instrumental views on friendship [48], and they tend to consider those with whom they like to play most to be their best friends. Accordingly, notions such as intimacy or similarity of attitudes still play a less important role in explaining the friendships of young children [48], and so it is not so surprising that ToM is not associated with peer popularity in this age group. This finding is also consistent with results showing that, in preschoolers, only empathy (but not ToM) is related to disruptive behavior [49].

ToM skills were assessed using a multitude of different measures, in particular in Study 1 that assessed mindreading skills with standard first-order tasks [36], as well as a comprehensive battery that assesses the diverse aspects of advanced ToM that develop during the elementary school years [33]. Accordingly, Study 1 used a broad range of measures; yet none of these tasks with developmental sensitivity within different age groups revealed significant findings, suggesting that the null result was not due to measurement issues.

Peer popularity was—contrary to expectation [26]—not related to self-esteem in both groups of children (global self-esteem). This finding is surprising, as it suggests that peer popularity may matter less for children’s and young adolescents’ self-esteem than previously thought. That is, in middle childhood, perhaps being considered well-liked by their peers may not necessarily make the child happy with their sense of self or bring them personal happiness, as relations with parents remain important at this age [22,26].

For the young adolescents in Study 2, the results suggest that perceiving oneself as ‘smart’ or doing well academically in school may not necessarily be a factor for peer popularity, as past studies show that some children hold stereotypes against those who do well in school as unpopular peers [50]. In addition, it may well be that Germany and Canada are both countries where peer popularity is less important to children’s and adolescents’ notions of self-esteem, and there may be more of an emphasis on academic competence. Indeed, popularity may not be a protective factor that helps build a self in relation to the evolved needs of those stages of the life cycle, which are likely to be fed by sources other than the peer group (e.g., by adults, rewards, etc.). This interpretation, however, needs to be followed up in future research.

In both studies, gender did not play a role in ToM, self-perceptions, or peer popularity. This finding is contrary to expectation, as gender differences are reported for popularity [51] and ToM [52], with females typically outperforming males. Also, there were no age-related changes in ToM performance in Study 2. Already 10-year-olds performed quite well on the ToM measure used in Study 2, suggesting that perhaps a plateau phase is reached with this instrument in early adolescence. Future research should explore this issue in more depth.

For educators and the school context, our findings suggest that ToM can be a protective factor for peer popularity and self-esteem, as it may allow especially older children to build meaningful relationships with others and to construct a firm understanding of the self. Several training programs exist for ToM in the classroom [53,54], and these produce significant effects for children’s ToM, even when they are given by teachers. Accordingly, classroom activities that help to strengthen mindreading skills such as perspective-taking and emotion recognition may be a fruitful way to help children in their everyday social lives [12], and indeed, studies have shown that such trainings can help to reduce children’s feelings of loneliness [55].

## 5. Limitations and Future Directions

The findings of the present studies should be considered in light of the limitations that they present. First, ToM was assessed using different measures across the two studies. Therefore, the findings are not directly comparable. Future studies should use the same tasks across contexts. Considering the low value of Cronbach’s alpha, it is possible that young children (5–8 years) may have struggled with the self-rating items, due to their developing self-concept and limited understanding of self-esteem. At this age, their perceptions of self-worth, especially regarding appearance and abilities, are still forming. This could have contributed to the suboptimal reliability of the scale. Future studies could address this aspect and use alternative instruments. Another limitation, aside from the potential difficulties in understanding the questionnaires (although clarification was always available), is the fact that information was collected only from the children. Future studies could explore these aspects by considering other key informants who are in contact with the children, as well as using observational methods. Future investigations could consider, in addition to self-esteem, the sense of self-efficacy, particularly in relation to tasks specific to the life stage being investigated, to examine possible connections.

In addition, the sampling of the present study with its convenience sampling does not allow generalizing to the broader population. Future work should draw nationally representative samples by using international cohort studies, which allow for comparison. Future studies could also investigate the role of teacher and parental support to examine changes in the model.

It is important to note that the two samples in our study were drawn from different cultural contexts—Germany in Study 1 and Canada in Study 2—which may have influenced the observed associations among ToM, self-perceptions, and peer popularity. Cultural factors, including language, educational practices, classroom composition, and social norms, can shape children’s self-concept and peer relations, as well as their responses to ToM assessments [56,57,58,59,60]. For instance, certain dimensions of self-esteem and the value placed on peer popularity may vary across cultural settings, potentially affecting both the measurement and interpretation of these constructs. Future research should further investigate the moderating role of culture to better understand how these variables interact in diverse sociocultural contexts and to ensure that our findings are interpreted with appropriate sensitivity to cultural influences.

## 6. Educational Implications

The present study showed a significant link between ToM and self-perceptions across two samples of children in middle childhood (aged 5 to 8 years) and early adolescence (aged 10 to 12 years). Peer popularity was related to ToM only in the older but not younger sample, and no significant associations emerged between peer popularity and children’s self-perceptions (self-esteem). This study highlights the importance of analyzing the antecedents of peer popularity in a developmental perspective that includes social and cognitive aspects. With regard to educational implications, these findings highlight the need for curricula to be culturally informed and developmentally sensitive, adapting to the cognitive and social growth of children at different ages, and integrating peer dynamics more explicitly as children progress through school. In particular, an interesting aspect that emerges is the implementation and structuring of support programs. These programs should focus on teaching methods and providing opportunities for students to develop perspective-taking and emotional literacy both in simple and complex situations. Together with the ability to manage communicative and interactive exchanges, these skills are essential for well-being and positive peer relationships. Therefore, it is simportant for educational institutions to prioritize these opportunities for their students, so that they can live in a positive environment that not only focuses on achieving academic goals but also on deeper aspects related to the higher educational goals of identity formation. Thus, it is essential to focus on actions that strengthen self-esteem and socio-relational skills, starting from the individual and their cognitive resources.

## Figures and Tables

**Table 1:** Measures Used in Studies 1 and 2.

	*First-Order ToM*	*Advanced ToM*		
		Social Reasoning	Reasoning About Ambiguity	Recognizing Transgressions of Social Norms
Study 1	Wellman scale	Higher-order false belief (3 items); double bluff task from the strange stories (1 item); eyes test (5 items)	multiple interpretations tasks (5 items)	Faux-pas recognition test (3 items); persuasion task from the strange stories (1 item)
Study 2	n/a	Socially ambiguous stories (2 stories, 7 items)	n/a	n/a

**Table 2:** Descriptive Results (Means and Standard Deviations) for Age Differences (Study 1).

	Kindergarten	Grade 1	Grade 2	
	5-year-olds	7-year-olds	8-year-olds	*F*
*First-Order ToM (% correct)*				
Wellman scale	62.70 (22.86)	77.50 (20.83)	83.60 (20.10)	17.23 ***
*Advanced ToM* (% correct)				
Social reasoning	30.70 (17.94)	46.73 (21.63)	56.75 (22.74)	26.18 ***
Reasoning about ambiguity	32.24 (34.10)	42.68 (34.11)	52.31 (34.77)	5.65 **
Recognizing transgressions of social norms	56.34 (30.88)	67.53 (26.80)	72.32 (22.15)	6.24 **
Self-esteem (0–4)	2.70 (0.68)	3.31 (0.52)	3.35 (0.46)	30.60 ***
Peer Popularity (*z*-values, −1.65–3.68)	1.52 (1.76)	1.89 (2.00)	2.32 (2.02)	2.81

Notes: ** *p* < 0.01; *** *p* < 0.001.

**Table 3:** Correlations among variables (Study 1).

	1	2	3	4	5	6
1. Social Reasoning	1.00					
2. Reasoning about ambiguity	0.28 ***	1.00				
3. Recognizing transgressions of social norms	0.24 ***	0.20 **	1.00			
4. Theory of mind, first order (Wellman scale)	0.45 ***	0.23 ***	0.36 ***	1.00		
5. Self-esteem	0.17 *	0.05	0.12	0.20	1.00	
6. Peer Popularity	0.08	−0.05	−0.09	0.03	0.08	1.00

Notes. * *p* < 0.05, ** *p* < 0.05, *** *p* < 0.001.

**Table 4:** Descriptive Results (Means and Standard Deviations) for Grade Levels (Study 2).

	Grade 4	Grade 5	Grade 6	*F*
	10-year-olds	11-year-olds	12-year-olds	
ToM (possible range 0–42)	27.43 (5.90)	28.30 (6.20)	28.62 (5.66)	0.50
*SPPC (self-esteem)*				
Academic competence	17.05 (4.60)	17.87 (4.22)	17.97 (4.50)	0.99
Social acceptance	18.23 (3.85)	18.38 (5.22)	18.55 (4.50)	0.07
Athletic competence	17.41 (4.41)	18.23 (4.65)	18.55 (4.51)	1.28
Physical appearance	18.61 (4.63)	18.82 (4.30)	18.68 (4.63)	0.40
Behavioral conduct	18.83 (4.30)	18.44 (4.24)	18.16 (4.06)	0.49
Global self-esteem	19.66 (3.54)	19.60 (4.20)	20.08 (3.92)	0.34
Peer Popularity (*z*-values)	2.96 (0.71)	2.94 (0.83)	2.91 (0.74)	0.06

**Table 5:** Correlations among Variables (Study 2).

	1	2	3	4	5	6	7	8
1. Theory of mind	1.00							
2. Academic competence	0.18 *	1.00						
3. Social acceptance	−0.03	0.46 ***	1.00					
4. Athletic competence	0.11	0.50 ***	0.39 ***	1.00				
5. Physical appearance	0.08	0.45 ***	0.39 ***	0.40 **	1.00			
6. Behavioral conduct	0.13	0.25 **	0.15 #	0.15 #	0.31 **	1.00		
7. Global self-esteem	−0.00	0.53 ***	0.50 ***	0.36 ***	0.71 ***	0.43 ***	1.00	
8. Peer popularity	0.22 **	−0.03	−0.04	−0.13	−0.07	0.11	−0.11	1.00

Notes. # *p* < 0.1, * *p* < 0.05, ** *p* < 0.005, *** *p* < 0.001.

**Table 6:** Summary of the Models (Study 2).

	Peer Popularity
Model 1	b (SE)
ToM	0.24 (0.09) **
Academic competence	−0.06 (0.09)
Gender	0.09 (0.08)
Grade level	−0.05 (0.08)
*R* ^2^	0.07
Model 2	b (SE)
ToM	0.31 (0.30)
Academic competence	0.02 (0.30)
Gender	0.10 (0.08)
Grade level	−0.05 (0.08)
ToM × academic competence	−0.12 (0.50)
*R* ^2^	0.06

Note: ** *p* < 0.01.

## Data Availability

Data can be requested directly from the corresponding author due to privacy reasons.
